# Patient Willingness to Consent to Mobile Phone Data Collection for Mental Health Apps: Structured Questionnaire

**DOI:** 10.2196/mental.9539

**Published:** 2018-08-29

**Authors:** Daniel Di Matteo, Alexa Fine, Kathryn Fotinos, Jonathan Rose, Martin Katzman

**Affiliations:** ^1^ The Centre for Automation of Medicine The Edward S Rogers Sr Department of Electrical and Computer Engineering University of Toronto Toronto, ON Canada; ^2^ START Clinic for Mood and Anxiety Disorders Toronto, ON Canada; ^3^ Department of Psychology Lakehead University Thunder Bay, ON Canada; ^4^ The Northern Ontario School of Medicine Thunder Bay, ON Canada; ^5^ Adler Graduate Professional School Toronto, ON Canada

**Keywords:** passive sensing, mobile phone sensing, psychiatric assessment, mood and anxiety disorders, digital privacy, mobile apps, mobile phone, consent

## Abstract

**Background:**

It has become possible to use data from a patient’s mobile phone as an adjunct or alternative to the traditional self-report and interview methods of symptom assessment in psychiatry. Mobile data–based assessment is possible because of the large amounts of diverse information available from a modern mobile phone, including geolocation, screen activity, physical motion, and communication activity. This data may offer much more fine-grained insight into mental state than traditional methods, and so we are motivated to pursue research in this direction. However, passive data retrieval could be an unwelcome invasion of privacy, and some may not consent to such observation. It is therefore important to measure patients’ willingness to consent to such observation if this approach is to be considered for general use.

**Objective:**

The aim of this study was to measure the ownership rates of mobile phones within the patient population, measure the patient population’s willingness to have their mobile phone used as an experimental assessment tool for their mental health disorder, and, finally, to determine how likely patients would be to provide consent for each individual source of mobile phone–collectible data across the variety of potential data sources.

**Methods:**

New patients referred to a tertiary care mood and anxiety disorder clinic from August 2016 to October 2017 completed a survey designed to measure their mobile phone ownership, use, and willingness to install a mental health monitoring app and provide relevant data through the app.

**Results:**

Of the 82 respondents, 70 (85%) reported owning an internet-connected mobile phone. When asked about installing a hypothetical mobile phone app to assess their mental health disorder, 41% (33/80) responded with complete willingness to install with another 43% (34/80) indicating potential willingness to install such an app. Willingness to give permissions for specific types of data varied by data source, with respondents least willing to consent to audio recording and analysis (19% [15/80] willing respondents, 31% [25/80] potentially willing) and most willing to consent to observation of the mobile phone screen being on or off (46% [36/79] willing respondents and 23% [18/79] potentially willing).

**Conclusions:**

The patients surveyed had a high incidence of ownership of internet-connected mobile phones, which suggests some plausibility for the general approach of mental health state inference through mobile phone data. Patients were also relatively willing to consent to data collection from sources that were less personal but expressed less willingness for the most personal communication and location data.

## Introduction

The assessment of mood and anxiety disorders is most commonly performed through clinician interview of the patient or patient self-report. These assessments question patients about their feelings and actions in situations throughout their daily life, asking patients to report the intensity and severity of their symptoms. A review by Meyer et al [1] found that although psychological and medical tests/assessments are valid overall, they have varying degrees of validity, from very low to very high, due to variations in assessment style and responses. Patients may be unwilling or unable to answer accurately due to inaccuracy of recollection [2], psychopathology and/or biases [3,4], ambiguity in the questions, or lack of comfort with their clinician, among other factors. Furthermore, the rating scales employed as part of these assessments are typically not objective—for example, asking the patient to rate their feelings of fear or anxiety, for which no objective measures exist.

An alternative to assessing mental disorders based on descriptions of feelings would be an assessment in which patient behaviors are observed and qualified as indicating increased or decreased severity of the symptoms of the patient’s mental disorder. Researchers have begun taking steps toward behavior-based assessments of mental disorders by employing mobile phones as sensing platforms to directly measure or otherwise infer behaviors. Mobile phones are an excellent tool for this purpose, since most owners carry them throughout their day [5] and a combination of sensor data and software techniques can be used to infer high-level behavior and contextual awareness [5-9]. Perhaps most importantly, a mobile phone offers the potential to collect objective data from the patient in contrast to the subjective information provided in interviews or self-reports. The data can be considered objective because there is no human interpretation or mediation of information that comes from sensors that directly measure physical properties. This objectivity of the measurement gives rise to the potential for objective assessment of mental health based on those data [10].

Prior research with this style of passively-sensed, mobile phone–based assessment has shown the ability to measure patients’ mental health states relating to disorders such as depression [11] and bipolar disorder [12,13]. Abdullah et al [12] used mobile phone data to assess the social rhythm metric, a marker of stability for individuals with bipolar disorder. They passively recorded geolocation, ambient light levels, communication activity, and ambient audio to infer the metric. Faurhollt-Jepsen et al [11] reported that they could classify the affective state of patients with bipolar disorder by analyzing features of the patients’ voices as recorded by the mobile phone app developed. Ben-Zeev et al [13] demonstrated that changes in depression severity were associated with changes in physical activity, speech duration, and sleep duration, all of which were measured passively through a mobile phone app. Place et al [15] generated behavioral indicators from mobile phone–collected data that were predictive of clinically assessed symptoms of depression and posttraumatic stress disorder [15].

While these techniques show promise as novel, objective assessment tools, they rely upon the collection and processing of large amounts of private data from a patient’s mobile phone. This presents several challenges when requesting patient consent to provide this data. Records of a patient’s whereabouts and communications could result in health care providers being subpoenaed for these records by law enforcement agencies. As a result, patients may not be willing to consent to wholesale collection of these data for fear of potential legal ramifications. Patients may also feel uneasy knowing that their health care providers have the ability to scrutinize their actions and communications on a very fine-grained level. This is a specific concern for patients with anxiety disorder. In particular, for those with social anxiety disorder, where a source of anxiety is the fear of judgment from others [16], personal data collected from a mobile phone could potentially form the basis of that judgment. Furthermore, a patient may also fear the possibility that their data could accidentally become public and reveal confidential thoughts, feelings, and behaviors or that perhaps there could be legal implications if the government were to have access to the data [17].

For the researchers and practitioners interested in designing, experimenting with, and deploying these kinds of mobile phone–based assessment tools, it will be important to have a sense of the patient population’s willingness to consent to the necessary data collection. A number of studies have surveyed the general consumer population to determine the adoption of Internet of Things [18-20] and wearable technologies [21,22] for health care purposes, and all clearly identified privacy concerns surrounding the data collected by these technologies. However, it is important to know specifically which sources of data available for collection on mobile phones are of most concern and therefore least likely to achieve consent. This question was also raised by Torous et al [23], who, in their study of patient interest in using mobile apps to monitor their mental health conditions, state that they did not address specifically to which sources of information patients would be willing to grant access. This information would allow researchers and developers to build systems that do not rely on unlikely-to-consent sources of data or to do extra work to find ways to address the concerns of patients on particular sources of data collection. It also gives insight into potential barriers that clinicians and researchers would need to address in order to deploy such systems in a health care setting.

The objectives of this research were as follows: first, since mobile phone–based assessment requires that patients own a mobile phone and use it regularly, it is necessary to measure the ownership rates of mobile phones within the patient population. We also seek to gauge the patient population’s willingness to enroll in research studies in which their mobile phone would be used as an experimental assessment tool for their mental health disorder. Finally, and most importantly, we seek to determine how likely patients would be to provide consent for each individual source of mobile phone–collectible data across the wide variety of potential data sources.

## Methods

### Participants and Procedure

Participants included 82 individuals referred to the START (Stress, Trauma, Anxiety, Rehabilitation, Treatment) Clinic for Mood and Anxiety Disorders, a tertiary care mood and anxiety disorder clinic for the management of their symptoms, located in the city of Toronto, Ontario, Canada. Male and female genders were equally represented with 46% (38/82) males, 46% (38/82) females, and 7% (6/82) individuals who did not respond to a question on gender. The average age of the participants was 41 (SD 14.0) years old. All new intakes and existing patients (ie, any patients in the waiting room) from August 2016 to October 2017 were recruited for the survey while waiting for their appointment with a clinician. Patients were asked to complete a pen-and-paper questionnaire designed to achieve the goals stated above. The questionnaire underwent ethics review by Optimum Clinical Research (protocol number WS2382578), and all respondents provided informed consent before completing the questionnaire.

### Materials

The questionnaire designed for this study consisted of 13 self-report questions. The introduction to the questionnaire provides context to the questions which follow by presenting the concept of mobile phone apps as a potential supplement or replacement to questionnaire-based methods of mental health assessment. It is explained that the collection and analysis of data from patients’ mobile phones may assist their clinicians in providing better care yet may also impact their privacy. The questions, therefore, are to survey people’s willingness to provide sources of information to clinicians and researchers in a scenario where a hypothetical mobile phone app were installed onto their personal mobile phone.

The questions are listed in Textbox 1. Questions 1 through 3 assess general mobile phone use, ownership information, and willingness to install an app that might help with mental health. Questions 4 through 12 ask respondents if they would be willing to share a specific source of data available on their mobile phone. The final question asks which specific brand of phone the respondent uses. For the 2 most potentially rich sources of information, SMS (short message service, or text) messages and raw ambient audio recordings made using the device’s microphone, multiple questions are posed in which the amount of information and granularity of the data collection are varied. For example, question 5 asks if respondents would allow collection of SMS metadata (which doesn’t contain the content of the message but surrounding information such as who the message was sent to/from and when message occurred), while question 6 asks respondents if they would allow analysis into the contents of their messages for the purposes of extracting potentially clinically relevant words.

Questions 10 through 12 are audio-related. Question 10, concerning the least detailed of the 3 audio data sources, asks respondents if they would share audio metadata, while question 11 concerns willingness to have speech recognition (word detection) performed upon their audio. Question 12 assesses willingness on the most detailed personal data: the unrestrained analysis of ambient audio.

### Analysis

Responses to questions 1 through 12 were coded as ordinal variables with 2 (yes and no responses) or 3 levels (yes, maybe, and no responses). When respondents chose to use the other categories of response and provided free-form text, these responses were interpreted as either a yes, maybe, or no response and coded accordingly. Responses to question 13 (on the phone brand type) were coded as a categorical variable. To test for correlation between responses to questions and respondent age, the Spearman rank correlation coefficient (Spearman ρ) was computed along with *P* values to test against the alternative hypothesis that the correlation was nonzero (using the exact permutation test). To test for associations between responses to questions and respondent gender, responses were cross-tabulated by gender and a chi-square test for independence was performed (2-tailed). All statistics and tests were computed using MATLAB software version 2014b (MathWorks).

**Survey questions.**

1. Do you own a mobile phone and use it daily?

(a) Yes, (b) Yes, but not daily, (c) No

2. Do you connect to the internet on your mobile phone, either using a mobile data plan or Wi-Fi?

(a) Yes, (b) No

3. Would you be willing to install and use a mobile phone app to help your doctor better diagnose mental health problems and/or provide treatment?

(a) Yes, (b) Maybe, but I would need to know more information first, (c) No, (d) Other—specify

4. Would you be willing to have the app collect and share your location? This would use your mobile phone’s Global Positioning System and would pinpoint your location on a map from time to time throughout the day.

(a) Yes (b) Maybe, but I would need to know more information first (c) No (d) Other—specify

5. Would you be willing to have the app record the number of contacts you call or send SMS (text messages) to and the dates and times when you phone or text them? The identities of your contacts would not be shared.

(a) Yes, (b) Maybe, but I would need to know more information first, (c) No, (d) Other—specify

6. Would you be willing to have the app read the contents of your text messages to look for keywords related to mental health? (For example, looking for the use of words like “tired,” “depressed,” or “happy.”) The whole text messages would not be shared, only detected keywords.

(a) Yes, (b) Maybe, but I would need to know more information first, (c) No, (d) Other—specify

7. Would you be willing to have the app record every time you create a calendar entry in your calendar app? The specifics of the calendar entry or event would not be shared, only the date and time that you create or modify it.

(a) Yes, (b) Maybe, but I would need to know more information first, (c) No, (d) Other—specify

8. Would you be willing to have the app record every time you turn your phone’s screen on or off (using the power or lock button)?

(a) Yes, (b) Maybe, but I would need to know more information first, (c) No, (d) Other—specify

9. Would you be willing to have the app use its sensors to try and detect if and when you are walking, running, in a car, or standing still?

(a) Yes, (b) Maybe, but I would need to know more information first, (c) No, (d) Other—specify

10. Would you be willing to have the app occasionally turn on and use the phone’s microphone to record the sounds of your surroundings? This audio would be used by the app to try and classify your surrounding as loud, quiet, or busy, but it would not attempt to recognize any words that you or people around you speak nor would it be listened to by humans. It would not record your phone calls, only ambient audio from time to time when you are not making a phone call.

(a) Yes, (b) Maybe, but I would need to know more information first, (c) No, (d) Other—specify

11. Consider the same scenario as question 10, but in this case the app will also detect and recognize specific words being spoken aloud by you or anyone else present in the recording. The app would attempt to recognize specific keywords like “tired,” “depressed,” or “happy.” This speech recognition would be done in software by the app, and the audio will never be listened to by humans.

(a) Yes, (b) Maybe, but I would need to know more information first, (c) No, (d) Other—specify

12. Consider the same scenario as question 11, but in this case the app is also able to perform any software-based processing to the recorded audio in order detect things that may be relevant to your mental health. This processing (in whatever form) would be done in software by the app, and the audio recordings will never be listened to by humans, nor will humans ever read a transcript of the recordings.

(a) Yes, (b) Maybe, but I would need to know more information first, (c) No, (d) Other—specify

13. What type of phone do you use daily? If you use multiple phones (work and personal), please select whichever corresponds to your personal phone.

(a) iPhone, (b) Android, (c) Blackberry, (d) Windows Mobile phone, (e) Other—specify

## Results

Of the 82 respondents, 73 (89%) reporting owning a mobile phone and using it daily (question 1). Rates of internet usage are also high, with 85% (70/82) of respondents reporting that they connect to the internet using their mobile phone (question 2). All respondents reported owning either iPhone, Android, Blackberry, or Windows Mobile mobile phones; Apple iPhones constituted 45% (35/78) of all mobile phones, Android devices constituted 57% (37/78), Blackberry constituted 6% (5/78), and Windows Mobile the remaining 1% (1/78) (question 13). Regarding question 3, willingness to install and use a mobile phone app to help their doctor better diagnose mental health problems and/or provide treatment, 41% of respondents (33/80) indicated that they would be willing to install and use such an app, with another 43% of respondents (34/80) indicating that they may be willing to use such an app, provided they were given more information about how the app functioned. Only 16% respondents (13/80) indicated absolute unwillingness to use such an app. Table 1 presents how responses to these questions (questions 1, 2, 3, and 13) correlate with respondent age and how the responses are associated with respondent gender.

**Figure 1:**
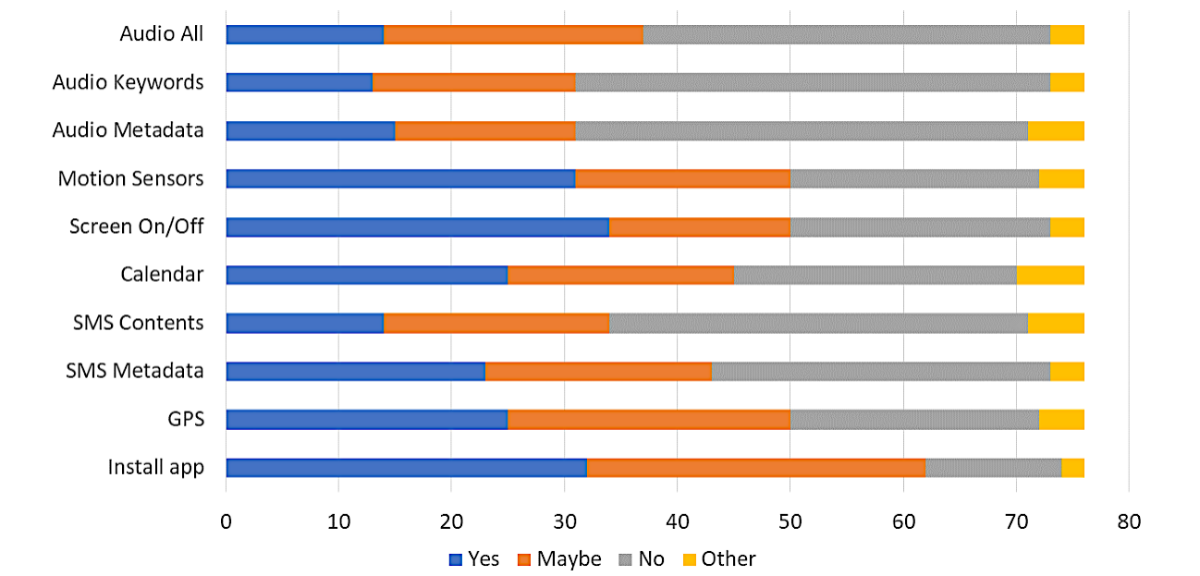
Respondent willingness to install app and grant permissions. GPS: Global Positioning System; SMS: short message service.

Survey questions 4 through 12 asked respondents if they would be willing to grant the hypothetical mental health monitoring app permission to collect a variety of data sources from their mobile phone. The responses corresponding to each permission (data source) are presented in Table 2. Figure 1 provides a graphical representation of this data, along with respondent willingness to install the app. It is worth noting that nearly none of the survey questions were strongly correlated with respondent age or associated with respondent gender. There is a weak positive correlation, however, between willingness to consent to audio recording and respondent age.

**Table 1:** Correlation with respondent age and association with respondent gender for mobile phone statistics and general willingness to install a mental health monitoring app.

Question topic	Correlation with respondent age		Association with respondent gender	
	ρ	*P* value	*Χ* ^2^_2_	*P* value
Mobile phone ownership (Q1)	0.08	.50	2.81	.24
Internet-connected mobile phone usage (Q2)	–0.09	.46	1.58	.21
Mobile phone types owned (Q13)	–0.10	.43	1.52	.68
Respondent willingness to install a mental health monitoring app (Q3)	0.10	.44	1.86	.39

**Table 2:** Survey respondent willingness to grant permission by category.

Permission	Willing to grant permission, n (%)				Correlation with respondent age			Association with respondent gender	
	Yes	Maybe	No	ρ		*P* value	*Χ* ^2^_2_		*P* value
GPS^a^ location (Q4)	28 (35)	26 (33)	26 (33)	–0.07		.60	0.18		.91
SMS^b^ metadata (Q5)	24 (30)	22 (28)	34 (43)	0.13		.31	1.00		.61
SMS contents (Q6)	16 (20)	21 (27)	42 (53)	0.02		.13	6.30		.04
Calendar (Q7)	26 (33)	26 (33)	27 (34)	–0.03		.83	4.73		.09
Screen on/off (Q8)	36 (46)	18 (23)	25 (32)	0.00		.97	0.48		.79
Motion sensors (Q9)	33 (42)	20 (26)	25 (32)	–0.04		.76	1.18		.55
Audio metadata (Q10)	16 (20)	18 (23)	46 (58)	0.25		.04	4.58		.10
Audio keywords (Q11)	14 (18)	20 (25)	46 (58)	0.29		.02	2.25		.33
Audio unrestrained (Q12)	15 (19)	25 (31)	40 (50)	0.32		.01	4.67		.10

^a^GPS: Global Positioning System.

^b^SMS: short message service.

## Discussion

### Principal Findings

Mobile phone ownership rates in the patient population surveyed in this study are high, with 89% of respondents reporting that they own a mobile phone and use it daily and 85% of patients connect to the internet with it. These results indicate that, in general, lack of mobile phone ownership itself will not present a barrier to the use of mental health monitoring apps in the future. This is further evidenced by American [24] and Canadian [25] mobile phone ownership statistics.

The vast majority of respondents reported owning either an iPhone (45%) or an Android device (47%). This corroborates surveys conducted by Gartner in 2018 [26] which indicate that Android and iOS, collectively, comprise 99.8% of the mobile phone operating systems. As each platform can require significant effort to support, this is relatively good news that suggests that development solely on iOS and Android platforms is sufficient to support the vast majority of patients.

We observe that overall respondents’ reception toward the notion of installing a mental health monitoring app is generally positive, with 84% answering either yes or maybe when asked if they would install and use such an app on their mobile phone. This finding shows that there is a general positive interest in using mobile phone apps to aid in mental health assessment. Considering that the questionnaire expresses that such an app is believed to help both clinicians and patients but makes no claims about proven effectiveness nor does it quantify said effectiveness in any way, it is plausible that if such apps are to be developed and proved to be effective that rates of adoption may be higher than reported in this study once patients are presented with these findings.

Respondent willingness to provide access to particular sources of data varies from a minimum of 43% answering yes or maybe in the case of audio metadata or keyword extractions (questions 10 and 11) to a maximum of 68% answering yes or maybe in the case of screen state (question 8). We feel that these results are encouraging considering the survey does not allay any potential fears that may be held by the respondents with regard to data security or data access. In a production-ready app, data security is likely to be a considerable point of focus, with efforts to encrypt data in transit and at rest. Furthermore, it may be possible to develop automated software algorithms to process much of the raw data and report higher level statistics of interest to clinicians [27,28], addressing patient fears that others would be scrutinizing them personally by, for example, reading their text messages or listening to their audio recordings. As our survey did not address any of these points, it may be possible that responses would be more positive given these guarantees.

It is worth noting that data sources that are low resolution in terms of providing personal or private information, such as screen state (Q8) and motion sensor data (Q9), are the most likely to be granted. Contrast this with sources of data that offer much more insight into a person’s private life, such as the contents of SMS messages (Q6) and unrestrained audio recording and analysis (Q12), which are among the least likely to be granted (only 20% and 19% yes responses, respectively). This could be interpreted as evidence toward the hypothesis that fear of scrutiny is responsible for unwillingness to provide access to data. If that hypothesis was supported, then this suggests that any automated analysis of data that removes humans from direct observation of the source data may be a method to improve patient willingness to provide data access. Further research is required to explore this hypothesis, however.

### Comparison With Other Studies

The rates of mobile phone ownership and use measured in this work is roughly in line with previous research in other areas. The results reported in this study are most similar to those measured in the United States, with a high 70% [29] to 80% range [30]. The predominance of Android and iOS devices within the population under study is also in line with current market statistics [26]. The mobile phone ownership and use measured in this study was somewhat lower than the 96% rate measured by Zhang et al [31] in a Chinese population.

While existing work demonstrates that mobile phone and/or wearable-based health assessment tools are being adopted despite concerns around privacy [21,22], the authors are unaware of any studies that have attempted to determine precisely which sources of data cause most concern. In the broader field of mobile phone apps in general (ie, without a focus on mental health care apps), Felt et al [32] found that SMS messages were the data source that was most cause for concern, more so than Global Positioning System (GPS) location. While they did not consider audio recordings, the identification of SMS messages being more invasive of privacy than GPS location is in line with our results. A similarly broad study into mobile phone user perceptions of privacy and security found that older users exhibited more privacy and security concerns [33]. This is in contrast with the population studied in this work, as we have measured a positive relationship between age and willingness to consent to audio recordings (ie, older people are more willing to consent to audio collection).

### Limitations

One limitation of the study is the small, concentrated population of respondents. All respondents presumably live within the Toronto area, and it is not clear how these results may generalize to the greater Canadian population or beyond. Another limitation of the study is the survey design. It is clear that respondents, in general, require more information to provide a sense of their willingness to provide access to data, as the proportion of maybe responses averaged across questions 4 through 12 was nearly one-third of respondents (28%). As mentioned earlier, 2 key pieces of information that would help respondents make more informed decisions are the effectiveness of the app in helping to monitor or manage their mental health if the data are provided and the risks involved and the steps being taken to protect the patient’s privacy. Conveying both of these pieces of information to prospective app users will be a challenge for health care providers willing to employ mobile mental health apps.

Another fundamental limitation of this study is one inherent to surveys in general: it is not clear that survey respondents who expressed interest in the hypothetical app and willingness to consent to collection of their data through the app would actually consent in a real-life scenario involving real mental health apps. We surmise this would depend upon how effective the apps were shown to be and what patient perception of the risks would be.

Finally, it would be interesting for future work to determine if there were any measurable differences between how patients with different disorders might consent to access to their data. One interesting hypothesis to test would be whether patients with social anxiety disorder, who fear evaluation and scrutiny, would be less likely to provide data access, possibly due to the fear of observation or judgment from others characteristic of the disorder [34].

### Conclusion

Given the potential for mobile technology to help patients monitor their mental health symptoms in a passive and pervasive way, it is helpful for researchers to understand how patients may respond to requests for access to their personal data. General interest in such an app is moderate, with 41% of respondents indicating they would install a monitoring app and 43% of respondents indicating they may install such an app. Willingness to provide data collection across different sources ranges from 18% to 46%, with more intrusive or private sources of data being more likely to be withheld. Finally, we support previous findings [24,25] that show mobile phone technology adoption alone will not pose a significant problem to fielding mental health apps, as 85% of respondents reported using an internet-connected mobile phone daily.

## Abbreviations

GPS: Global Positioning System
SMS: short message service
START: Stress, Trauma, Anxiety, Rehabilitation, Treatment
